# Editorial for the Special Issue—“Recent Advances of Novel Pharmaceutical Designs for Anti-Cancer Therapies”

**DOI:** 10.3390/ijms24098238

**Published:** 2023-05-05

**Authors:** Bernhard Biersack

**Affiliations:** Organische Chemie 1, Universität Bayreuth, Universitätsstrasse 30, 95440 Bayreuth, Germany; bernhard.biersack@uni-bayreuth.de or bernhard.biersack@yahoo.com

Cancer is one of the leading causes of death worldwide, despite the promising developments in terms of the curing and management of certain cancer types that have occurred over the last decades saving and prolonging the lives of numerous patients. Severe side effects, as well as intrinsic and acquired cancer drug resistance, pose constant problems, which lead to inefficient therapy outcomes and consequently to worse prognoses for affected patients. Although the development of a new anticancer drug is costly and takes much time, it is necessary to identify new drug candidates, which can overcome the drawbacks of the existing arsenal of cancer medicines, and which can become new treatment options for hitherto untreatable and/or highly aggressive cancer types.

The Special Issue “Recent Advances of Novel Pharmaceutical Designs for Anti-cancer Therapies” was launched in the autumn of 2021 in order to cover outstanding anticancer projects and to unveil new developments and breakthroughs in terms of pharmaceutical drug and target discovery. Eminent scientists working in the field contributed innovative methods, targets, and new interesting hit-and-lead candidates. At this point, eleven research and review articles were published in this Special Issue, and the contents of nine of them are briefly summarized in the following. For more detail, I strongly recommend reading the original full length open access articles published so far.

Zhou et al. described the synthesis and antitumor activities of new bis-cinnamoyl derivatives, which were prepared by simple methods from inexpensive starting materials [1]. The most promising compound I_23_ was active against three cancer cell lines in the low micromolar concentration range. Mechanistically, I_23_ induced apoptosis, stopped tumor cell migration, and stabilized microtubules.

The anticancer activity of the natural phenol pterostilbene was investigated by Wawszczyk et al. in melanotic and amelanotic melanoma models [2]. The described anti-melanoma effects of pterostilbene were mediated by p21, cyclin D1, and caspase-3. Interestingly, pterostilbene induced apoptosis in amelanotic melanoma cells in a caspase-dependent way, while apoptosis in melanotic melanoma cells remained unaffected by pterostilbene. These tumor-specific effects of pterostilbene deserve more profound investigation.

Curcumin is another prominent natural phenolic compound with promising anticancer properties, and Bhattacharyya et al. studied the effects of the fluorinated semi-synthetic curcumin derivative CDF and its new formulation with 2-hydroxypropyl-β-cyclodextrin (CDFHCD) on pancreatic ductal adenocarcinoma (PDAC) [3]. The CDFHCD formulation displayed increased stability and water solubility while conserving the anti-PDAC activity of CDF. Thus, the formulated CDF derivative CDFHCD appears to be a promising anticancer drug for clinical trials with PDAC patients in the future.

Adenosine deaminases acting on RNA (ADAR) are RNA-editing enzymes with relevance for various human diseases including cancer. ADAR2 was identified as a promising target for the design of inhibitors, and computer chemistry techniques (molecular docking and molecular dynamics simulation) were applied to identify potential ADAR2 inhibitors [4]. Various natural polyphenols, bis-naphthoquinones, and the well-known dye indigo were found to be promising ADAR2 inhibitor candidates, which should be considered for further studies on their anticancer properties as ADAR2 inhibitors.

Metal complexes are a promising class of anticancer compounds, and platinum-based drugs hold a salient position in the treatment of various solid tumors. Complexes of other metals were also found to be highly active against tumors. Schleser et al. prepared and evaluated a series of new iodidogold(I)-NHC (*N*-heterocyclic carbene) complexes in esophageal adenocarcinoma (EAC) cells [5]. The most active gold complexes of this study exhibited high antiproliferative activity, induced apoptosis, suppressed *c*-Myc and cyclin D1 expression, and inhibited EAC colony and spheroid formation. 

The identification and validation of new cancer drug targets is of great importance for the development of new innovative anticancer drugs. Rotermund et al. studied the expression of carbonic anhydrases IX and XII in colorectal cancers, as well as the potential of these vital enzymes as possible drug targets [6]. Four consensus molecular subgroups were established based on their differing carbonic anhydrase expression states, and the response to treatment with the inhibitor SLC-0111 was clearly associated with target expression in colorectal cancer spheroids of these subgroups. 

The specific targeting of the translation machinery of proliferating tumor cells is a promising strategy to fight cancer. Steinmann et al. identified the eukaryotic initiation factor 4A1 (eIF4A1) as a prognostic factor in hepatocellular carcinoma (HCC), whose molecular function can be suppressed by treatment with inhibitors [7]. These eIF4A1 inhibitors led to high antiproliferative and strong pro-apoptotic effects in HCC, and the anticancer activities of the eIF4A1 inhibitors were significantly augmented by the combination with a pan-mTOR inhibitor. 

In addition to these original research manuscripts, three review articles were published in this Special Issue. One of them was provided by Zhang et al., which also deals with a promising target of the cellular translation machinery, the eukaryotic elongation factor 1A (eEF1A) [8]. Among the described eEF1A inhibitors, plitidepsin is already approved for multiple myeloma treatment, and another compound (metarrestin) is currently in clinical trials. In addition, further natural and synthetic eEF1A inhibitors with sound anticancer effects were described and discussed.

Finally, a comprehensive review on the application of the proteolysis-targeting chimera (PROTAC) strategy for the treatment of cancer was published in this Special Issue, which prompted a high interest among the readers of the International Journal of Molecular Sciences [9]. Bifunctional conjugates of small molecule target protein binders with E2 ligase binding scaffolds led to pronounced anticancer activities and remarkable curing effects. It is noteworthy that this strategy also holds considerable promise for the treatment of other human diseases. 

All in all, this Special Issue covers a well-balanced collection of state-of-the-art topics in the prospering and competitive field of anticancer drug design and development, which can address and inspire oncologists, biologists, and medicinal chemists among the readership of this journal. 
